# Using Machine-Learning and Network Analysis to Investigate the Risk Factors of AI Dependence: The Crucial Role of Escape and Social Motivation

**DOI:** 10.3390/bs16050772

**Published:** 2026-05-14

**Authors:** Yufan Chen, Xiaoyin Miao, Zeyang Yang

**Affiliations:** Department of Psychology, School of Education, Soochow University, Suzhou 215123, China; 2302405026@stu.suda.edu.cn

**Keywords:** AI dependence, machine learning, network analysis, escape motivation, social motivation, social anxiety

## Abstract

People have become accustomed to studying or working with the guidance of artificial intelligence (AI) in recent years. Studies have begun investigating the risk factors of AI dependence, though most have used hypothesis-testing methods. The present study aimed to investigate predictors of AI dependence using machine-learning and network analysis, which are data-driven approaches. The included risk factors were Big Five personality traits, self-efficacy, depression, social anxiety, adverse childhood experiences, and AI use motivation, selected based on theories and empirical studies. Participants consisted of 1258 university students (942 females and 316 males) with a mean age of 22.11 years (SD = 2.69). Four machine-learning algorithms were tested, including Elastic Net, Random Forest, XGBoost, and LightGBM. Machine-learning results indicate that escape and social motivation for AI use, along with social anxiety, were the main predictors of AI dependence. Network analysis results show that escape and social motivation were the most central nodes, with the highest Expected Influence (EI) indices. This study indicates that when addressing mental health problems related to AI dependence, it is more effective to focus on emotional isolation and social interaction challenges rather than simply cutting down on AI use.

## 1. Introduction

In recent years, artificial intelligence (AI) has experienced rapid, unprecedented growth, accompanied by widespread adoption (Avicena Tech Corp., 2024; Hassani et al., 2020; Heimberger et al., 2024; Cao & Cai, 2025). Generative AI, exemplified by applications such as ChatGPT, has gained increasing academic interest (Ciudad-Fernández et al., 2025; Yankouskaya et al., 2025). Several studies have begun to focus on the dependent, problematic, or addictive use of AI (Al-Obaydi & Pikhart, 2026; Hu et al., 2023; Huang et al., 2024; Montag & Elhai, 2025). Empirical evidence suggests that users may develop excessive reliance on AI systems, driven by features such as anthropomorphic interaction, personalised feedback, and instant gratification (Yankouskaya et al., 2025). These patterns resemble characteristics of behavioural addiction, including poor self-control over usage, withdrawal symptoms, and conflicts in daily lives (Yankouskaya et al., 2025).

The use of the term “addiction” in the context of AI continues to be debated. Recent studies have warned against overpathologising normal interactions with emerging technologies (e.g., Ciudad-Fernández et al., 2025). Instead, “AI dependence” has been used to describe overuse or reliance on AI technologies, with possible negative effects (Huang et al., 2024). AI dependence refers to patterns of excessive reliance on AI suggestions or interactions, especially in completing tasks and managing emotions (Huang et al., 2024; Goh et al., 2025). This concept differs from, but is connected to, “reliance behaviours,” which encompass a wider range of cognitive and collaborative patterns in human-AI interaction (Hou et al., 2026). This reliance may indicate a shift in AI’s role from a supportive tool to a more central part of users’ cognitive and psychological processes, with potential risks including reduced critical thinking and negative mental health effects (Yankouskaya et al., 2025). Accordingly, the present study adopts the term “AI dependence” to enable a more precise and theoretically grounded investigation of the risk mechanisms underlying human-AI interaction. The present study uses the term “AI dependence” rather than “AI addiction” or “problematic AI use” to avoid the risk of overpathologising AI use as an addiction and of misinterpreting problematic use as unethical use of AI technology. Unlike “AI addiction”, which denotes a clinically defined disorder, “AI dependence” is used in the present study to describe potentially maladaptive use of AI that can lead to negative consequences (mental health risks or conflicts) (Huang et al., 2024).

The Interaction of Person-Affect-Cognition-Execution (I-PACE) model explains the early and later developmental stages of addictive behaviours, such as gaming disorder, gambling disorder, and compulsive buying (Brand et al., 2019). It indicates that addictive behaviours result from dynamic interactions between individuals’ core characteristics and affective, cognitive, and executive processes in specific situations (Brand et al., 2019). In this framework, a person’s core characteristics serve as key distal predictors of addictive behaviours, including biopsychological constitution, psychopathology, personality, social cognition, childhood experiences, and specific motives for use (Brand et al., 2019). Recent studies often employed the I-PACE model as the theoretical foundation in emerging digital contexts, indicating it could help understand problematic use of AI technologies (Hu et al., 2023; Huang et al., 2024).

Self-Determination Theory (SDT) offers a theoretical framework for understanding human motivation and behavioural regulation (Deci & Ryan, 1985). It distinguishes between intrinsic and extrinsic motivation and posits that the degree of motivational internalisation and behavioural engagement is directly influenced by the satisfaction of three basic psychological needs (i.e., autonomy, competence, and relatedness) (Deci & Ryan, 1985). Within this framework, competence is closely linked to self-efficacy, which is defined as an individual’s belief in their ability to complete tasks (Bandura, 1997). Motivation determines the direction and persistence of behaviour (Deci & Ryan, 1985). Empirical evidence suggests that higher self-efficacy increases individuals’ propensity to rely on specific technologies by enhancing their perceived controllability and utility (L. Zhang & Xu, 2024). In contrast, extrinsic or compensatory motivation may lead to excessive or maladaptive patterns of technology use (Goh et al., 2025). Therefore, self-efficacy and motivation can be regarded as key psychological factors shaping technology use patterns. Within the realm of AI, these factors may further support the development of problematic behaviours or dependence (Goh et al., 2025; L. Zhang & Xu, 2024).

The Theory of Planned Behaviour (TPB) posits that behaviour is primarily determined by behavioural intention, which predicts and explains individual behaviour in specific contexts. Behavioural intention is jointly influenced by behavioural attitude, subjective norm, and perceived behavioural control; it reflects an individual’s level of motivational readiness to perform a specific behaviour and is regarded as the most direct predictor of behaviour (Ajzen, 1991). Motivation is manifested through positive attitudes and behavioural intentions, which, in turn, influence behavioural engagement and persistence (Montag & Elhai, 2025). Existing research indicates that this model demonstrates strong explanatory power in technology usage behaviours (such as smartphone dependence) (Yue et al., 2020). Recent studies show that strong usage motivation can enhance behavioural intention, thereby increasing usage frequency and dependence, and consequently promoting the development of problematic AI use behaviours (Huang et al., 2024).

For theoretical coherence, the present study integrates the I-PACE model, the SDT, and the TPB into a unified explanatory framework. The I-PACE model serves as an overall framework for including personal characteristics variables as predictors of AI dependence. SDT can serve as a theoretical basis for exploring the motivational mechanisms that drive AI engagement or dependence. TPB further explains how motivation influences behavioural intentions. Overall, these theories provided a comprehensive framework for understanding the development of AI dependence.

Recent studies have identified several key factors associated with AI dependence, including personality, self-efficacy, mental health conditions, and motivation for AI use (Estrada-Araoz et al., 2025; Hu et al., 2023; Huang et al., 2024; Kırcaburun & Özdemir, 2026; Zhong et al., 2024). Individuals with high levels of neuroticism, who are likely to be affected by academic stress, tend to overly rely on AI technology to avoid facing academic insecurities and emotional distress (Zhong et al., 2024). In a sample of Peruvian university students, a negative correlation was observed between academic self-efficacy and AI dependence (Estrada-Araoz et al., 2025). S. Zhang et al. (2024) reported that individuals with low academic self-efficacy were more likely to experience higher levels of academic stress. These individuals had higher expectations of AI technology, which ultimately resulted in greater AI dependence. Unlike the internet addiction-mental health studies, Huang et al. (2024) did not find a bidirectional relationship between mental health problems and AI dependence. Instead, mental health problems demonstrated a long-term, one-way effect on AI dependence. Escape motivation and social motivation also showed a mediating effect between mental health problems and AI dependence (Huang et al., 2024). Meanwhile, Hu et al. (2023) found that social anxiety, as another key personal characteristic, was positively linked to AI dependence through loneliness and rumination. Moreover, studies have shown that adverse childhood experiences (ACEs) are a critical risk factor for Internet addiction (Chaudhary et al., 2024; Hao et al., 2024), which was also noted in the I-PACE model (Brand et al., 2019). However, evidence of the link between ACE and AI dependence remains limited, and further studies are required.

Overall, multiple factors may be linked to AI dependence, as indicated by the empirical and theoretical studies mentioned above. However, because research on AI dependence is limited, the links between AI dependence and these factors are based on limited empirical evidence or theoretical frameworks and require further evaluation and validation. It is therefore essential to further test whether these factors are actual risks of AI dependence and to compare their predictive significance. The present study aimed to investigate the mechanisms underlying how multiple personal characteristics (Big Five personality traits, self-efficacy, depression, social anxiety, ACE, and AI use motivations) influence individuals’ AI dependence. The selection of these potential risk factors is based on the I-PACE model (Brand et al., 2019) and empirical studies as discussed above. More specifically, since the I-PACE model clearly suggests that predictors of addictive behaviours can include personal core characteristics (e.g., early childhood experience, coping, and psychopathology), cognitive and affective responses, craving, self-control, etc., the present study determined to include personality, self-efficacy, depression, social anxiety, ACE, and motivation in accordance with the I-PACE framework.

Rather than testing theory-constrained causal paths or hypotheses, this research adopted a data-driven approach combining machine-learning and network analysis. Machine-learning models were used to identify the most important predictors of AI dependence. Network analysis further visualised and quantified the structural relationships among all variables and identified the most central variables. The present study aims to use a data-driven approach to examine the theoretically proposed risk and protective factors associated with AI dependence. While existing studies have identified the correlates of AI dependence, less attention has been paid to how the predictive factors are organised within a topological system. Thus, the present study also explores the structural roles of the key variables using network analysis.

Therefore, research questions are proposed as follows:

RQ1: Which personal characteristics (Big Five personality traits, self-efficacy, depression, social anxiety, ACE, and AI use motivation) most strongly predict AI dependence as identified by machine-learning models?

RQ2: What are the most central nodes in the network of AI dependence and the personal characteristics (Big Five personality traits, self-efficacy, depression, social anxiety, ACE, and AI use motivation)?

## 2. Methods

### 2.1. Participants

The study recruited 1344 Chinese university students through the survey platform www.naodao.com. Initially, a total of 1344 questionnaire responses were collected. After excluding responses with patterned responses (i.e., responding with the same response for all questions) and those that failed the attention check, 1258 participants were included in the present study (942 females and 316 males). The average age of all participants was 22.11 years (SD = 2.69). The participants consisted of 106 first-year students (8.4%), 230 second-year students (18.3%), 298 third-year students (23.7%), 327 fourth-year students (26.0%), and 297 postgraduates (23.6%). Postgraduates included both master’s and doctoral students. Detailed demographic information is presented in Table 1.

### 2.2. Measurements

#### 2.2.1. AI Dependence

The AI dependence scale developed by Huang et al. (2024) was used to assess the participants’ AI dependence. The scale includes five items (e.g., “I feel anxious when I am unable to use artificial intelligence”). Responses were rated on a 4-point Likert scale, scoring from 1 (strongly disagree) to 4 (strongly agree). The Cronbach’s alpha was 0.78.

#### 2.2.2. Chinese Big Five Personality Inventory Brief Version (CBF-PI-B)

The Chinese Big Five Personality Inventory brief version (CBF-PI-B), a 15-item self-report scale derived from the original 40-item version, was used to assess Neuroticism, Extraversion, Openness, Agreeableness, and Conscientiousness (X. Zhang et al., 2019). Items were rated on a 6-point scale (1 = strongly disagree, 6 = strongly agree). The Cronbach’s alpha was 0.86 for Neuroticism, 0.88 for Extraversion, 0.91 for Openness, 0.84 for Agreeableness, and 0.67 for Conscientiousness.

#### 2.2.3. General Self-Efficacy Scale (GSES)

The General Self-Efficacy Scale (GSES) was developed by Schwarzer and Jerusalem (1995). This study used the Chinese version validated by C. Wang et al. (2001). The scale consists of 10 items to assess an individual’s confidence in coping with challenges (e.g., “I can always find a solution to a problem.”). Each item is rated on a 4-point Likert scale (1 = not at all true, 4 = exactly true). The Cronbach’s alpha was 0.91.

#### 2.2.4. Patient Health Questionnaire-9 (PHQ-9)

The Patient Health Questionnaire-9 (PHQ-9) (Kroenke & Spitzer, 2002), a brief self-report instrument based on the DSM-IV diagnostic criteria, was used to assess depressive symptoms. The Chinese version demonstrated good reliability and validity (W. Wang et al., 2014). Participants were asked to report the frequency of their symptoms over the past two weeks. Each of the nine items is rated on a 4-point Likert scale, ranging from 0 (not at all) to 3 (nearly every day). Higher total scores indicate greater severity of depressive symptoms. The Cronbach’s alpha was 0.87.

#### 2.2.5. Social Anxiety Scale

The Chinese version of the Social Anxiety Scale, revised by J.-L. Wang et al. (2011) was used to assess the social anxiety levels of participants. The scale consisted of five items (e.g., “I feel shy in front of strangers”) rated on a 5-point Likert scale, ranging from 1 (not at all characteristic of me) to 5 (extremely characteristic of me). However, preliminary reliability analysis indicated that the item “I only talk with specific people” exhibited low internal consistency with the other items. This discrepancy was likely due to semantic ambiguity, which introduced measurement noise. Removing this item significantly improved the scale’s internal consistency, with Cronbach’s alpha increasing from 0.55 to 0.81. Consequently, this item was excluded from the final analysis.

#### 2.2.6. Adverse Childhood Experiences (ACEs)

Following the literature (Lin et al., 2021; T. Zhang et al., 2023), we extracted 12 indicators of Adverse Childhood Experiences (ACEs) from the CHARLS study, including seven conventional (physical abuse, emotional neglect, household substance abuse, household mental illness, domestic violence, incarcerated household member, and parental separation or divorce) and five extended metrics (unsafe neighbourhood, peer bullying, parental death, sibling death, and parental disability). Each indicator was dichotomised (0 vs. 1), and a cumulative score ranging from 0 to 12 was calculated for each participant. Based on these cumulative scores, participants were further categorised into five subgroups: 0, 1, 2, 3, and 4 or higher.

#### 2.2.7. AI Use Motivation Scale

Motivation for AI use was assessed using the 12-item scale developed by Huang et al. (2024). The instrument comprises four distinct dimensions: AI escape motivation, AI social motivation, AI instrumental motivation, and AI entertainment motivation. A sample item is, “I use AI to search for or obtain the information I need.” Participants responded on a 4-point Likert scale, ranging from 1 (strongly disagree) to 4 (strongly agree). The Cronbach’s alpha values for the four subscales were 0.82 (escape), 0.87 (social), 0.69 (instrumental), and 0.84 (entertainment).

#### 2.2.8. Demographic Information

The data analysis incorporated several demographic variables, including gender, age, being an only child, grade, father’s education, mother’s education, and family location. Prior research suggests that these factors may significantly influence individuals’ attitudes toward artificial intelligence (Sun & Zhou, 2025).

### 2.3. Procedure

Data were collected via the Naodao platform (www.naodao.com). This study adhered to the principles of the Declaration of Helsinki. All participants were provided with a consent form at the beginning of the survey and voluntarily participated in this study. Upon completing the survey, participants received a 1 CNY reward. The collected raw data underwent subsequent cleaning and processing for analysis.

### 2.4. Data Analysis

The present study used a machine-learning approach rather than traditional hypothesis-testing methods. Machine-learning algorithms are suitable for analysing high-dimensional data with potentially complex and nonlinear relationships. Given that many potential predictors were included, machine learning would be useful for identifying the key predictors of AI dependence and their relative importance as a data-driven framework. The initial aim of the present study is exploratory rather than hypothesis testing. Four machine-learning algorithms, including Elastic Net, Random Forest, XGBoost, and LightGBM, were used to detect the key predictors of AI dependence. Multiple machine-learning algorithms are compared and selected, and the models are evaluated through cross-validation. In this study, 80% of the data was allocated as the training set, and the remaining 20% was designated as the testing set, with a fixed random seed of 123 to enhance reproducibility. For the Elastic Net model, predictors were transformed into matrix format. The mixing parameter alpha was tuned across a grid from 0 to 1 in increments of 0.1 using 10-fold cross-validation. The final model was selected based on the optimal alpha and the lambda value that minimised cross-validated error. For the Random Forest model, the number of trees was set to 300, mtry was defined as the square root of the total number of predictors, and the terminal node size was set to 5. For both XGBoost and LightGBM, model performance was optimised using 5-fold cross-validation with early stopping to identify the optimal number of boosting iterations.

Performance of the four machine-learning algorithms was evaluated using root mean square error (RMSE), mean absolute error (MAE), and coefficient of determination (*R*^2^) metrics. Lower RMSE and MAE values, alongside higher *R*^2^ values, indicate superior predictive capability of the models. The SHAP (Shapley Additive exPlanations) algorithm was used to quantify the contribution value of each predicting variable to the degree of AI dependence, and the key factors that significantly affect AI dependence were screened out.

Network analysis was adopted to detect the most central or influential node in the network of AI dependence and the other variables. The network was estimated using the EBICglasso method with a tuning parameter (γ) set to 0.5, which balances model sparsity and sensitivity. Expected Influence (EI) indices were calculated to estimate the centrality of the nodes. All data analyses were conducted using R version 4.5.0.

## 3. Results

### 3.1. Descriptives, Correlations, and Linear Regression

**Figure 1:**
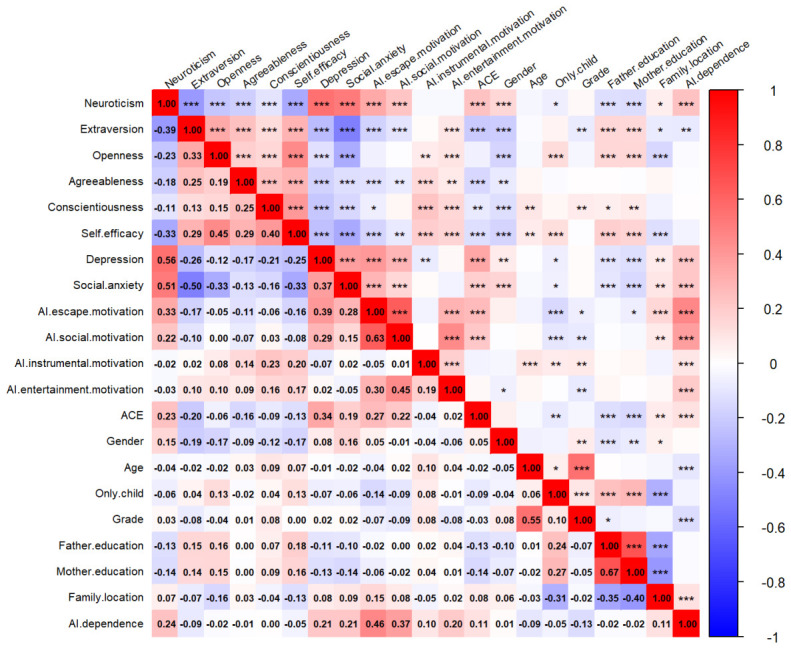
Pearson’s correlations. *Note*. * *p* < 0.05, ** *p* < 0.01, *** *p* < 0.001. *N* = 1258.

The descriptive statistics for each variable are shown in Table 2. The total scores of each variable are normally distributed, and the Cronbach’s α coefficients indicate good reliability. Pearson’s correlation coefficients are shown in Figure 1. AI dependence was significantly positively correlated with neuroticism, depression, social anxiety, AI use motivations, and ACE. AI dependence was negatively correlated with extraversion, age, and grade. Table 3 shows the linear regression results. Neuroticism, social anxiety, AI escape motivation, AI social motivation, and AI instrumental motivation positively and significantly predicted AI dependence. 

### 3.2. Machine Learning

**Figure 2:**
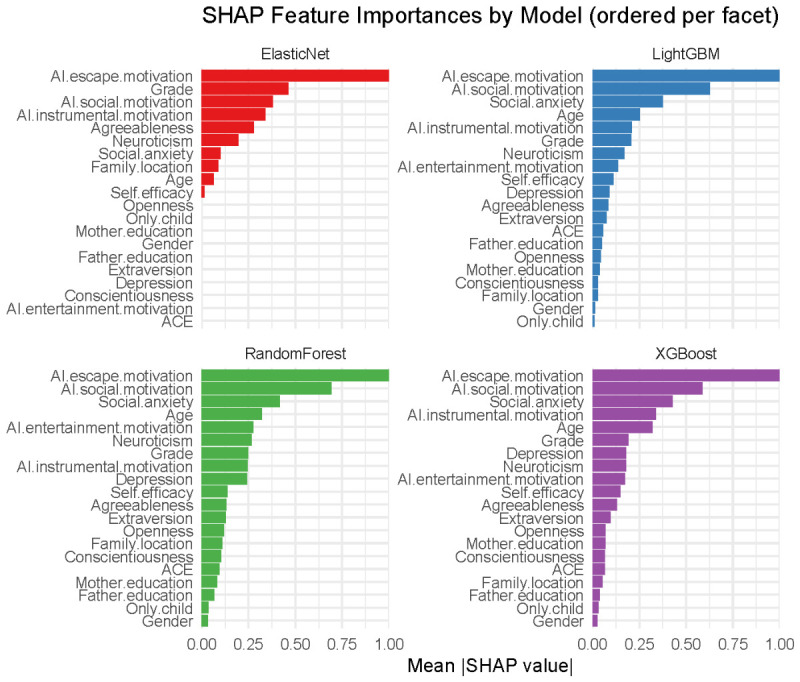
SHAP feature importances by model (ordered per facet).

To obtain a powerful prediction model for AI dependence, the present study employed four machine-learning algorithms, namely Elastic Net, Random Forest, XGBoost, and LightGBM. Figure 2 shows the SHAP feature importance rankings for different models. Overall, AI use motivation (particularly escape and social motivation) and social anxiety emerged as key predictors of AI dependence.

**Figure 3:**
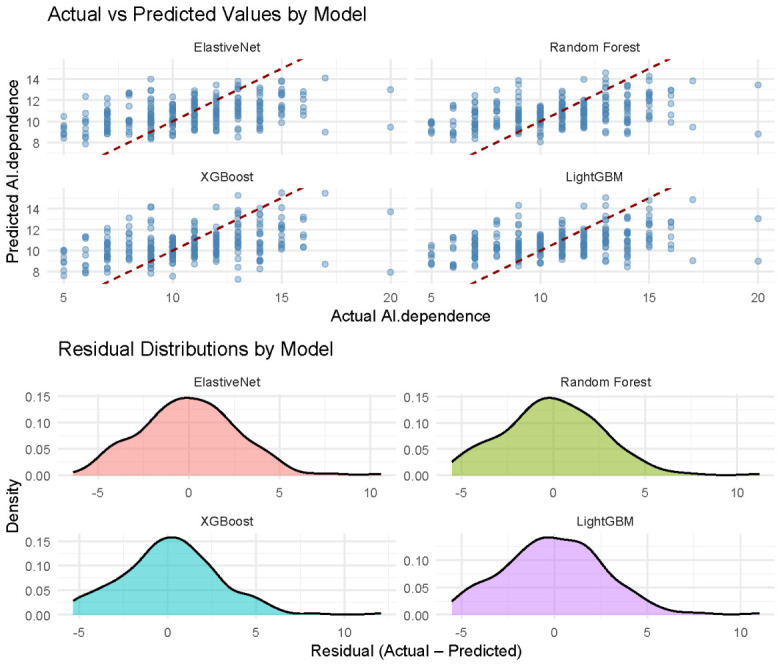
Model Prediction Accuracy and Residual Distributions.

As shown in Table 4, the three tree-based ensemble models (Random Forest, XGBoost, and LightGBM) demonstrated high goodness-of-fit on the training set. However, their performance on the test set dropped sharply. The substantial discrepancy between the performance metrics on the training and test sets indicates overfitting. Thus, these models are unsuitable for identifying stable and universal predictors. The regularised linear model, Elastic Net, exhibited comparatively greater robustness and generalisation capability, as reflected in the smaller performance gap between the training and test sets. On the test set, it achieved a modest coefficient of determination (*R*^2^ = 0.17) on the test set, along with lower RMSE and MAE values. This indicates that, among all models, the Elastic Net model provides the best explanatory power for the variance related to AI dependence and has achieved the optimal balance between avoiding overfitting and maintaining predictive accuracy. Figure 3 shows the models’ prediction accuracy and residuals. The point cloud of the actual and predicted AI dependence, and the residual distributions (approximately centred at zero with relatively symmetrical shapes), indicate good accuracy and reliable performance of the models.

### 3.3. Network Analysis

**Figure 4:**
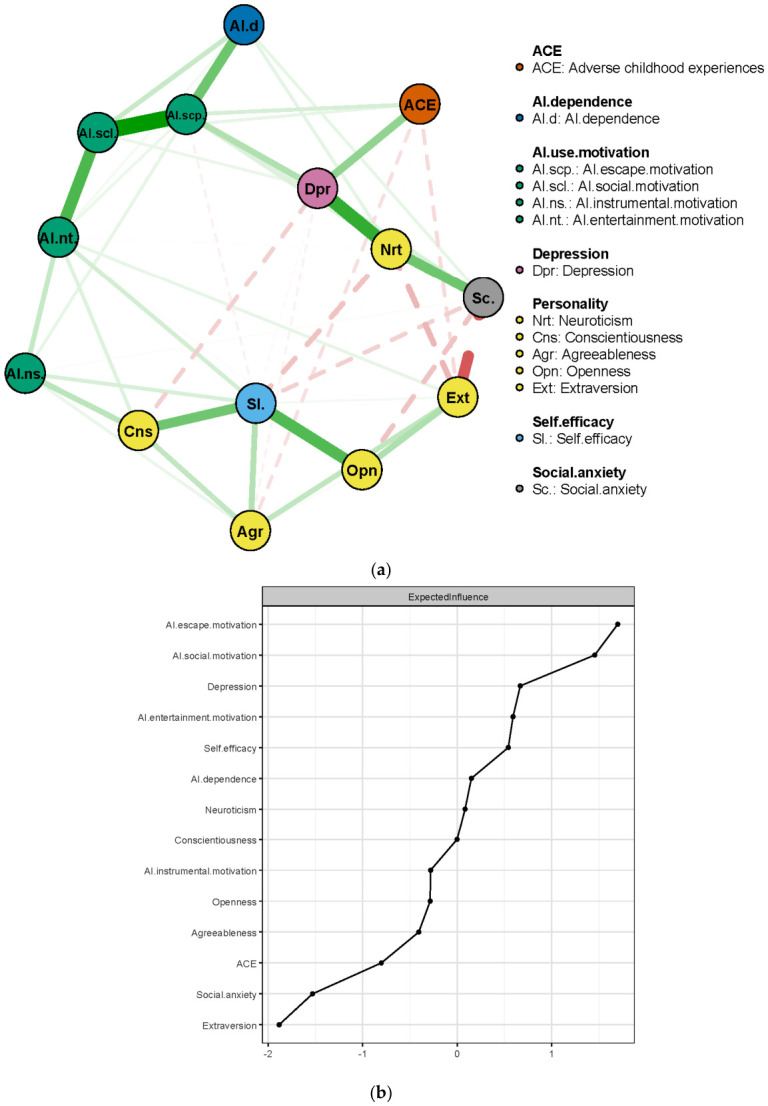
Network structure (**a**) and Expected Influence index (**b**).

Network analysis was conducted to evaluate the connections among AI dependence, potential predictors, and to identify the most central or influential variables. The network structure is presented in Figure 4a. The mean weight of the network was 0.035. The number of non-zero edges was 51 out of 91. The strongest associations in the network were observed between AI social motivation and AI escape motivation, suggesting a close relationship between these motivations for AI use.

**Figure 5:**
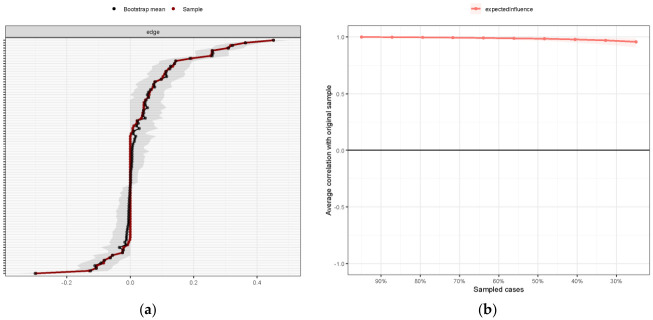
Network accuracy and stability. *Note*. (**a**) Edge weights and bootstrapped confidence intervals. The y-axis represents the edges. The red line indicates the estimated edge, while the dark area indicates the 95% bootstrap confidence interval. (**b**) Stability of Expected Influence by the case-dropping bootstrapping approach. The red line indicates the variation trend of the centrality correlation between subsamples and the original sample as the subsample sampling rate changes.

The Expected Influence metric was used to estimate the node centrality, as shown in Figure 4b. Escape and social motivation for AI use were the most central nodes in the network with the highest Expected Influence, indicating that they are highly connected to the other variables in the network. It is noteworthy that Expected Influence reflects the relative connectedness between the nodes rather than causal effects. They should be interpreted as the importance indicators rather than directional effects. Figure 5 shows that the network had good accuracy and stability. The Expected Influence CS coefficient was 0.75, suggesting good stability and robustness of the central nodes.

## 4. Discussion

### 4.1. Summary of the Findings

The present study examined the risk factors of AI dependence using data-driven approaches. Machine-learning models reveal that AI use motivation (particularly escape and social motivation) and social anxiety are key factors influencing AI dependence. Network analysis shows that escape and social motivation are the central nodes, supporting the findings from the machine-learning analysis.

### 4.2. Theoretical and Practical Implications

Escape and social motivation for AI use were the top predictors of AI dependence across all four machine-learning models, as shown in the correlation and regression results with significance. It demonstrates the crucial predictive role of motivation on behaviours, as discussed in the SDT and TPB frameworks. Such results support the previous study’s findings that escape and social motivation significantly predicted subsequent AI dependence (Huang et al., 2024). It suggests that individuals’ dependence on AI is primarily motivated by a desire to escape daily problems and the need for social interaction to ease loneliness. Such results are consistent with previous studies, which found that technology use can be seen as a social crutch for escaping social problems (Fullwood et al., 2017; Yang et al., 2021). Huang et al. (2024) reported that AI escape and social motivations acted as mediators between mental health risks (depression and anxiety) and AI dependence. The results of network analysis in the present study supported their findings from a data-driven perspective. AI escape and social motivations had the highest EI values, emphasising their central roles within the entire network. It seems that individuals’ AI dependence was directly linked to escapism and social motivations for using AI. While these motivations are not the root cause of the mechanism, they have complex connections with other issues (e.g., anxiety, depression) and personality, as shown by the correlations in the present study.

The present study identified that motivation can not only be a peripheral predictor of AI dependence but also play a central structural role in the network of AI dependence and correlates. In line with previous studies (Huang et al., 2024), it suggests that AI use motivation can serve as a bridge connecting emotional vulnerability (e.g., social anxiety), personality, cognitive tendencies (e.g., self-efficacy), and behavioural engagement with AI. Theoretically, this finding contributes to the understanding of the predictors of AI dependence by examining an integrated system in which motivation plays a key role. In other words, the contribution of the present study can not only identify predictors, but also reveal the structural centrality roles of predictors within the mechanism.

The present study identified several other key predictors of AI dependence, including social anxiety, neuroticism, and Grade level. Social anxiety consistently ranked among the top predictors in the machine-learning models. It also demonstrates significant relationships with AI dependence in both correlation and linear regression analyses. This aligns with previous findings that social anxiety significantly predicted AI dependence (Hu et al., 2023). Neuroticism was identified as another important predictor of AI dependence, as indicated by machine-learning models, correlation analyses, and regression analyses, aligning with previous studies (Zhong et al., 2024). These findings appear to support and clarify the predictive roles of personal core characteristics (e.g., personality and social anxiety) in behavioural addictions within the theoretical frameworks (Billieux et al., 2015; Brand et al., 2019). Interestingly, grade showed significant negative predictive effects on AI dependence and ranked second in the SHAP feature importances of the Elastic Net model, which demonstrated the highest explanatory power. It suggests that students at earlier stages of learning may rely more on AI than senior students. However, these results mainly suggest the predictive effects of negative factors. Previous evidence indicates that positive attitudes towards AI can increase problematic social media use (Montag & Elhai, 2025). Future studies should investigate whether positive factors, such as attitudes, perceived strength of AI use, or positive emotions, could predict AI dependence.

In addition, self-efficacy was not significantly correlated with AI dependence in the correlation or as a significant predictor in the linear regression analysis. It was not identified as a top predictor in the machine-learning models. S. Zhang et al. (2024) reported a negative and significant correlation between academic self-efficacy and AI dependence, but did not identify a significant direct effect in the mediation analysis. They found that academic stress fully mediated the relationship between them. Considering the results of the present study, the link between self-efficacy and AI dependence may be complex, and potential mediators such as stress should be further explored. General self-efficacy was measured because it shows a broad belief in a person’s ability to deal with challenges in different situations. Since AI use behaviours are part of many daily activities, a general measure is better than a specific one for capturing overall self-regulatory confidence.

The observed overfitting in the tree models (Random Forest, XGBoost, LightGBM) provides more insights into the data. It suggests that the tree models might have captured context-dependent patterns in the sample, rather than stable and generalizable relationships. It indicates that the underlying associations could be more complex and vary across different samples. Thus, a larger sample size might be necessary to detect the predictive roles of the variables in the present study. However, the regularised model (Elastic Net) might be more powerful, and the smaller set of predictors might be a more generalisable solution.

The findings of the present study have several tentative practical implications. The primary predictive effect of motivation for escape and social AI use suggests that the underlying drivers of AI-related behaviours may be more closely linked to users’ motivational processes than to usage intensity alone. More specifically, when addressing individuals’ problematic or dependent AI use situations, focus should be on the issues they aim to escape from, such as loneliness, social anxiety, or lack of social interactions in daily life. Therefore, when creating interventions to address excessive reliance on AI with adverse effects, the main focus might not be to eliminate AI use but to offer coping strategies for social support issues. Furthermore, the AI developers could consider the users’ escape and social motivations, as well as their social anxiety. For example, AI chatbot designers might include notes advising unusually heavy or dependent users to seek help from their friends in real social situations or through other channels, not just AI. Moreover, it is vital for students to improve their AI literacy and avoid excessive reliance on AI. For example, studies could examine how to reduce students’ thoughtless reliance on AI by offering interventions or guidance on critical thinking while solving problems (Hou et al., 2026). However, these implications should be interpreted with caution, as the present study employed only a cross-sectional design. Future studies could use longitudinal and experimental designs to explore the effectiveness of the potential strategies as noted above.

### 4.3. Limitations and Future Directions

The present study has limitations. Self-reported responses on questionnaires were collected. The participants may provide socially desirable responses or give biased answers due to memory loss. Therefore, future research should document individuals’ real AI usage behaviours and employ diverse methods to assess their possible dependence on AI. In addition, the sample in the present study might not cover the truly dependent individuals, though over one thousand participants were recruited. Future studies could employ different measures of AI dependence and attempt to identify individuals with extremely high AI dependence, which may lead to functional impairment in daily life. Furthermore, the current study only recruited university students as participants and overlooked other age groups. Future studies should include both younger children and elderly people as participants to determine whether AI dependence varies across age groups. Furthermore, future research could examine how people with varying levels of dependence engage with AI and the resulting impacts. Moreover, the gender imbalance of the sample might be a limitation, and the results should be interpreted with caution. Future studies could recruit a more representative sample with equal numbers of females and males to increase the generalisability of the findings.

## 5. Conclusions

The present study adopted data-driven approaches, including machine-learning and network analysis, to detect the key predictors of AI dependence. Results show that escape and social motivations, as well as social anxiety, are the main key predictors. It suggests that when addressing AI dependence-related mental health issues, it is better to focus on an individual’s emotional or affective isolation and social interaction difficulties in daily life rather than merely reducing their AI use.

## Figures and Tables

**Table 1:** Demographic information.

Variables	Categories	n	%
Gender	Male	316	25.1
	Female	942	74.9
Grade	First year	106	8.4
	Second year	230	18.3
	Third year	298	23.7
	Fourth year	327	26.0
	Master	274	21.8
	PhD	23	1.8
Only child	Yes	341	27.1
	No	917	72.9
Family location	Urban	747	59.4
	Countryside	511	40.6

**Table 2:** Descriptive statistics.

	Mean	SD	Min	Max	Skew	Kurtosis	Alpha
AI.dependence	10.8	2.86	5	20	0.19	−0.37	0.78
Neuroticism	11.11	3.45	3	18	−0.21	−0.43	0.86
Extraversion	9.28	3.68	3	18	0.35	−0.6	0.88
Openness	10.74	3.42	3	18	−0.16	−0.47	0.91
Agreeableness	13.05	2.64	3	18	−0.75	0.94	0.84
Conscientiousness	12.84	2.54	3	18	−0.3	0.01	0.67
Self-efficacy	25.26	5.75	11	40	0.2	−0.19	0.91
Depression	7.22	4.91	0	27	1.00	1.06	0.87
Social anxiety	13.81	3.57	4	20	−0.67	−0.06	0.81
AI.escape.motivation	5.72	2.11	3	12	0.5	−0.24	0.82
AI.social.motivation	5.79	2.33	3	12	0.59	−0.4	0.87
AI.instrumental.motivation	10.27	1.42	3	12	−0.66	0.76	0.69
AI.entertainment.motivation	7.83	2.23	3	12	−0.24	−0.53	0.84
ACE	1.46	1.52	0	10	1.4	2.34	-

*Note*. *N* = 1258.

**Table 3:** Linear regression results using AI.dependence as the dependent variable.

	B	β	*SE*	*t*	*p*	Fit
(Intercept)	3.33	NA	1.11	3.00	0.003 **	
Neuroticism	0.07	0.09	0.03	2.71	0.007 **	
Extraversion	0.02	0.03	0.02	0.85	0.396	
Openness	0.00	0.00	0.02	0.15	0.878	
Agreeableness	0.03	0.03	0.03	1.21	0.227	
Conscientiousness	0.00	0.00	0.03	0.02	0.984	
Self.efficacy	0.02	0.03	0.02	0.96	0.336	
Depression	0.00	0.00	0.02	0.07	0.944	
Social.anxiety	0.07	0.08	0.03	2.58	0.010 *	
AI.escape.motivation	0.46	0.34	0.05	9.99	0.000 ***	
AI.social.motivation	0.15	0.13	0.04	3.66	0.000 ***	
AI.instrumental.motivation	0.22	0.11	0.05	4.14	0.000 ***	
AI.entertainment.motivation	0.02	0.01	0.04	0.49	0.627	
ACE	−0.05	−0.03	0.05	−1.07	0.284	
Gender	−0.05	−0.01	0.17	−0.30	0.762	
Age	−0.05	−0.05	0.03	−1.51	0.130	
Only.child	0.22	0.03	0.17	1.27	0.204	
Grade	−0.19	−0.08	0.07	−2.79	0.005 **	
Father.education	−0.06	−0.02	0.09	−0.66	0.508	
Mother.education	0.10	0.04	0.09	1.10	0.272	
Family.location	0.36	0.06	0.16	2.26	0.024 *	
						*R*^2^ = 0.269 **
						95% CI[0.22, 0.30]

*Note*. * *p* < 0.05, ** *p* < 0.01, *** *p* < 0.001. *N* = 1258.

**Table 4:** Machine-learning model fit comparisons.

Model	*RMSE*	*MAE*	*R* ^2^
Elastic Net (Train)	2.4038	1.9400	0.2847
Elastic Net (Test)	2.6397	2.0923	0.1715
Random Forest (Train)	1.1093	0.8835	0.8477
Random Forest (Test)	2.6672	2.1076	0.1542
XGBoost (Train)	1.8866	1.5124	0.5594
XGBoost (Test)	2.7258	2.1536	0.1166
LightGBM (Train)	2.1677	1.7458	0.4183
LightGBM (Test)	2.6930	2.1356	0.1377

## Data Availability

The data are not publicly available due to privacy concerns.
